# Variability of MMP/TIMP and TGF-β1 Receptors throughout the Clinical Progression of Chronic Venous Disease

**DOI:** 10.3390/ijms19010006

**Published:** 2017-12-21

**Authors:** Pedro Serralheiro, António Novais, Elisa Cairrão, Cláudio Maia, Carlos M. Costa Almeida, Ignacio Verde

**Affiliations:** 1 Norfolk and Norwich University Hospital, Norwich NR47UY, UK; 2 Faculty of Health Sciences, CICS-UBI—Health Sciences Research Centre, University of Beira Interior, 6201-506 Covilhã, Portugal; a25155@fcsaude.ubi.pt (A.N.); ecairrao@fcsaude.ubi.pt (E.C.); cmaia@fcsaude.ubi.pt (C.M.); iverde@fcsaude.ubi.pt (I.V.); 3 Department of General Surgery (C), Coimbra University Hospital Centre, 3041-801 Coimbra, Portugal; c.m.costa.almeida@gmail.com; 4 Faculty of Medicine, University of Coimbra, 3000-548 Coimbra, Portugal

**Keywords:** chronic venous disease, matrix metalloproteinases (MMPs), tissue inhibitors of metalloproteinases (TIMPs), transforming growth factor (TGF)-β receptors, varicose vein, gene expression

## Abstract

Chronic venous disease (CVeD) is a prevalent condition with a significant socioeconomic burden, yet the pathophysiology is only just beginning to be understood. Previous studies concerning the dysregulation of matrix metalloproteinases (MMPs) and their inhibitors (tissue inhibitors of metalloproteinases (TIMPs)) within the varicose vein wall are inconsistent and disregard clinical progression. Moreover, it is highly plausible that MMP and TIMP expression/activity is affected by transforming growth factor (TGF)-β1 and its signaling receptors (TGFβRs) expression/activity in the vein wall. A case–control study was undertaken to analyze genetic and immunohistochemical differences between healthy (*n* = 13) and CVeD (early stages: *n* = 19; advanced stages: *n* = 12) great saphenous vein samples. Samples were grouped based on anatomic harvest site and subjected to quantitative polymerase chain reaction for *MMP1*, *MMP2*, *MMP8*, *MMP9*, *MMP12*, *MMP13*, *TIMP1*, *TIMP2*, *TIMP3*, *TIMP4*, *TGFβR1*, *TGFβR2*, and *TGFβR3* gene expression analysis, and then to immunohistochemistry for immunolocalization of MMP2, TIMP2, and TGFβR2. Decreased gene expression of *MMP12*, *TIMP2*, *TIMP3*, *TIMP4*, and *TGFβR2* was found in varicose veins when compared to controls. Regarding CVeD clinical progression, two facts arose: results across anatomical regions were uneven; decreased gene expression of *MMP9* and *TGFβR3* and increased gene expression of *MMP2* and *TIMP3* were found in advanced clinical stages. Most immunohistochemistry results for tunica intima were coherent with qPCR results. In conclusion, decreased expression of TGFβRs might suggest a reduction in TGF-β1 participation in the MMP/TIMP imbalance throughout CVeD progression. Further studies about molecular events in the varicose vein wall are required and should take into consideration the venous anatomical region and CVeD clinical progression.

## 1. Introduction

Matrix metalloproteinases (MMPs) are a large family of endopeptidases that are secreted in their latent form by different cells in the venous wall (including fibroblasts, vascular smooth muscle cells, and leukocytes) and have proteolytic activities that participate in cellular homeostasis, adaptation, and tissue remodeling [1,2,3]. They are known for degrading collagen, elastin, and other extracellular matrix (ECM) macromolecules present in the structure of many tissues [1,2], and may affect other cellular processes including endothelium-mediated dilation, vascular smooth muscle cell migration and proliferation, as well as modulation of Ca^2+^ signaling and contraction in vascular smooth muscle [2,4]. MMP activity can be inhibited by four different tissue inhibitors of metalloproteinases (TIMP1–4) [1,2]. Tissue homeostasis is thus achieved by a tight balance of MMP and TIMP expression/activity. When this balance is disturbed, dysregulated MMP activities result—a fact associated with many diseases [3].

Transforming growth factor β (TGF-β) is a multifunctional growth factor that is widely expressed in diverse tissues, and which has critical and specific roles during embryogenesis and in maintaining the homeostasis of adult tissues [5]. This growth factor has three different isoforms in mammals (TGFβ1–3) [5,6]. TGF-β1 is secreted in a latent complex by different cell types (including leukocytes, platelets, and osteocytes) and is a potent chemo-attractant for inflammatory cells [7,8,9,10]. TGF-β1 signaling involves heteromeric complexes comprised of type I (e.g., transforming growth factor-β receptor 1 (TGFβR1)) and II (e.g., TGFβR2) transmembrane receptors, Smads (as signaling effectors, as well as transcription regulators), and non-Smad signaling pathways, leading to cell state-specific modulation of gene transcription [5,10]. TGFβR2 is capable of binding TGF-β1 alone, while TGFβR1 can only bind the ligand in cooperation with TGFβR2 [5,10]. TGF-β1 access to the signaling receptors is regulated by membrane-associated coreceptors (e.g., TGFβR3) that are thought to not signal directly [5,10].

Despite being intensively studied in the past decades, chronic venous disease (CVeD) is far from being completely understood. Theoretical explanations regarding venous pathophysiology and genesis of varicosities in the lower limbs vary. Recent explanations suggest that venous hypertension may induce leukocyte-endothelial activation and initiation of a series of inflammatory processes with alterations at a molecular level within the vein wall (e.g., production of free radicals, inflammatory mediators, and proteolytic enzymes) leading to dilated and tortuous veins, valvular incompetence, and blood stasis, which in turn promote the perpetuation of local venous hypertension [7,8,9,10,11,12,13,14,15,16]. A variety of molecules, such as TGF-β1, have been proposed to be involved in the regulation of leukocyte adhesion and recruitment [7,9,10,11,12,16,17]. Furthermore, a crucial role of MMP/TIMP imbalance in varicose vein formation and CVeD progression has been increasingly recognized [9,10,12,17,18,19,20,21,22,23,24,25,26,27,28]. Nevertheless, published results are not always consistent and show wide discrepancy among them (i.e., the same proteins were found to be increased, decreased, and even unchanged in varicose vs. non-varicose veins) [29].

We have previously demonstrated that TGF-β1 may directly intervene in the gene expression of MMP/TIMP in the great saphenous vein wall [30], which reinforced the hypothesis that the inflammatory process may lead to morphologic changes within the vein wall that is mediated by MMP and TIMP expression/activity [8,12,31]. Increasing knowledge about TGF-β1 signaling mechanisms and regulation indicates that signaling responses are extensively defined by TGF-β1 receptor availability and function [10,32]. It would, therefore, seem reasonable to expect that the effect of TGF-β1 on MMP and TIMP expression/activity depends on TGFβR expression/activity within vein wall cells.

Although the MMP/TIMP derangement in varicose veins is widely accepted, little is known about what triggers this imbalance and how it is related to TGF-β1 activity. Moreover, there are no studies associating MMP, TIMP, or TGFβR expression in vein wall cells with CVeD clinical progression. With the aim of achieving a better understanding on how MMP/TIMP and TGFβR gene expression and their presence vary within healthy/varicose venous walls, a cross-sectional case–control study was undertaken to analyze genetic and immunohistochemical differences between CVeD veins (grouped by clinical stages) and healthy veins.

## 2. Results

Table 1 summarizes the main demographic and clinical features of the participants. Ethnicity is not presented as all participants were Caucasian. In order to control demographic and clinical variability between groups, two subsamples of the 44 participants were used to study differences in gene expression among the control, CEAP2–3, and CEAP4–6 groups (*n* = 29) and between the CEAP2–3 and CEAP4–6 groups (*n* = 31). Therefore, differences regarding sex, age, BMI, and pregnancies presented *p* > 0.05.

### 2.1. MMP, TIMP, and TGFβR Gene Expression in Healthy and Varicose Vein Walls

**Figure 1:**
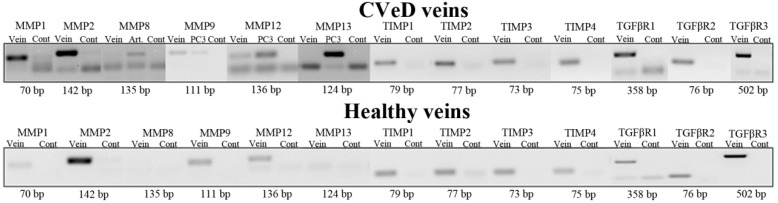
PCR analysis of several matrix metalloproteinases (MMPs), tissue inhibitors of metalloproteinases (TIMPs), and transforming growth factor-β receptors (TGFβRs) in cDNA pools of varicose and healthy veins. Amplification of β-actin housekeeping gene was used as a control of the cDNA synthesis. Umbilical arteries or the PC3 prostate cell line was used as a positive control for the amplification of *MMP8*, *MMP9*, *MMP12*, and *MMP13* in varicose veins.

PCR analysis confirmed the gene expression of all MMPs, TIMPs, and TGFβRs in the vein samples, except for *MMP8* and *MMP13* (Figure 1). The absence of *MMP8* and *MMP13* gene expression was reconfirmed after using umbilical arteries and the PC3 prostate cell line cDNA as positive controls. Also, *MMP1* and *TGFβR1* were excluded from further qPCR analyses due to very low gene expression in the cDNA pools.

**Figure 2:**
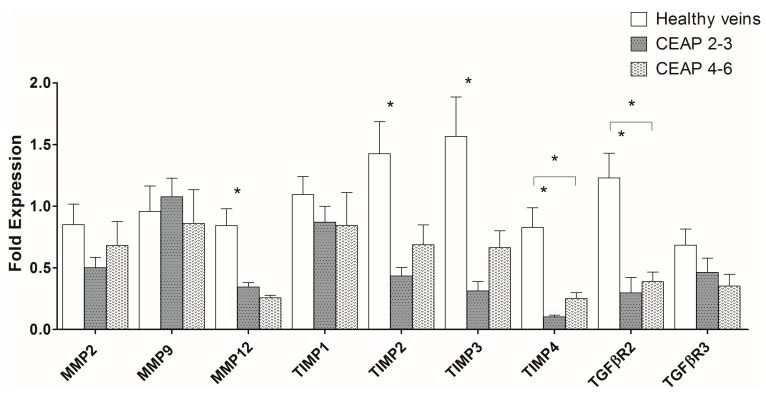
MMP, TIMP, and TGFβR gene expression in healthy, CEAP2–3, and CEAP4–6 veins (from the tibiotarsal junction). Their gene expression was determined by qPCR and after normalization with β-actin housekeeping gene. All results are expressed as fold expression. Error bars indicate mean ± standard error of the mean (SEM), *n*_(healthy)_ = 13, *n*_(CEAP2-3)_ = 10, and *n*_(CEAP4-6)_ = 6. * *p* ≤ 0.05.

Figure 2 presents the comparison between healthy and CVeD veins from the tibiotarsal region. The gene expression of *MMP12* (*p* = 0.006), *TIMP2* (*p* = 0.010), *TIMP3* (*p* = 0.026), *TIMP4* (*p* < 0.001), and *TGFβR2* (*p* = 0.001) was significantly decreased in the CEAP2–3 veins when compared to controls. Similarly, the gene expression of *TIMP4* (*p* < 0.001) and *TGFβR2* (*p* = 0.019) was significantly decreased in the CEAP4–6 veins when compared to controls.

**Figure 3:**
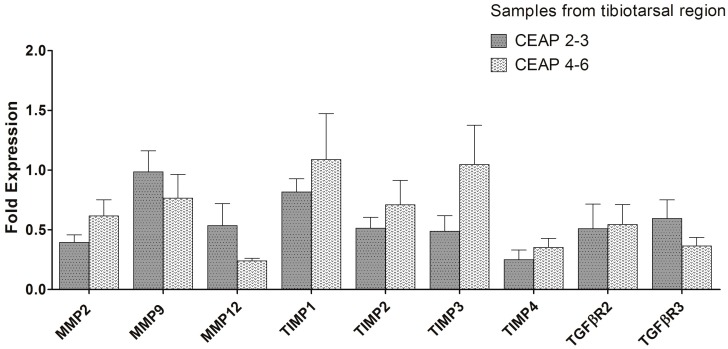

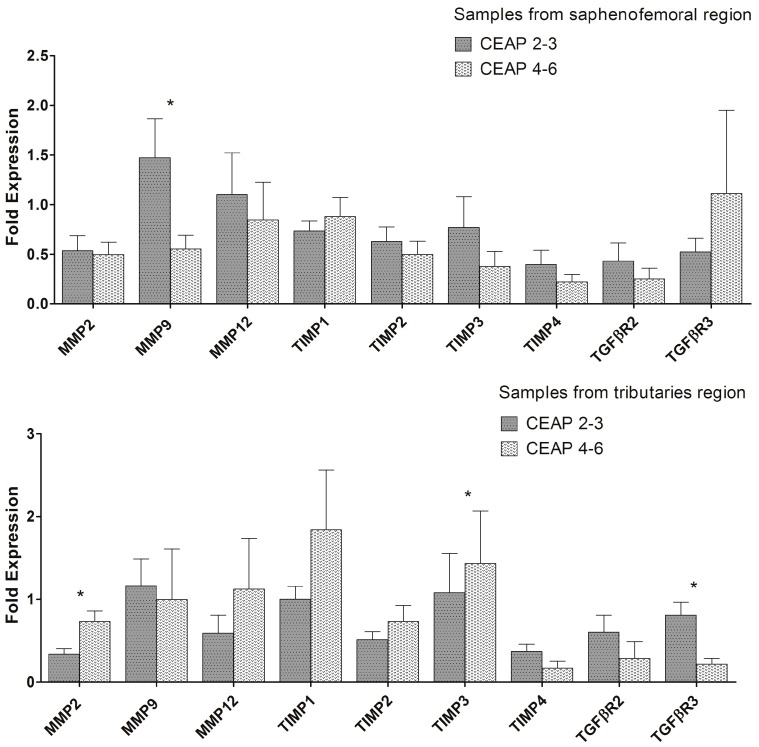
MMP, TIMP, and TGFβR gene expression in the CEAP2–3 and CEAP4–6 veins (from three different regions). Their gene expression was determined by qPCR after normalization with β-actin housekeeping gene. All results are expressed as fold expression. Error bars indicate mean ± SEM, *n*_(CEAP2–3)_ = 19 and *n*_(CEAP4–6)_ 12. * *p* ≤ 0.05.

Comparisons between the CEAP2–3 and CEAP4–6 vein groups (Figure 3) from different anatomic harvest sites (tibiotarsal, saphenofemoral, and tributaries) were also performed. No significant differences in MMP, TIMP, and TGFβR gene expression were found between the two CVeD groups from the tibiotarsal junction. From the saphenofemoral junction, only *MMP9* gene expression was significantly lower in the CEAP4–6 veins (*p* = 0.027). Finally, in varicose tributary veins, *MMP2* (*p* = 0.002) and *TIMP3* (*p* = 0.050) gene expressions were significantly increased, while *TGFβR3* gene expression (*p* = 0.002) was significantly decreased in the CEAP4–6 group.

### 3.2. MMP, TIMP, and TGFβR Immunoreactivity in Healthy and Varicose Vein Walls

**Figure 4:**
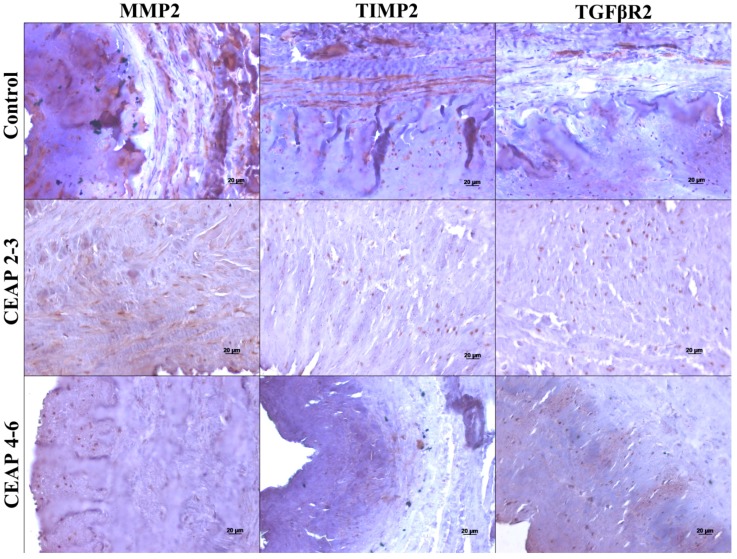
Immunodetection of MMP2, TIMP2, and TGFβR2 in healthy, CEAP2–3, and CEAP4–6 veins. Immunohistochemistry (IHC) was performed and revealed using a peroxidase substrate. Decreased labeling of proteins was observed in chronic venous disease veins. Scale bar represents 20 μm.

Qualitative results of IHC analysis are shown in Figure 4 and Table 2. Positive immunostaining for MMP2, TIMP2, and TGFβR2 was more consistently found in both intima and media layers of varicose and healthy veins. A closer look at the staining intensity revealed that MMP2 was decreased in all tunicae, TIMP2 was decreased in the intima and media, and TGFβR2 was slightly decreased in all tunicae of varicose veins when compared to controls.

From the comparisons between the CVeD groups, MMP2 was generally unchanged (except for the saphenofemoral junction samples where it was decreased in the intima and media of the CEAP4–6 group), TIMP2 was generally decreased in the media and adventitia but slightly increased in the intima (except for the saphenofemoral samples) of the CEAP4–6 group, and TGFβR2 appeared to have no relevant difference among the groups.

## 3. Discussion

This cross-sectional case–control study was set up in an attempt to resolve existing discrepancies and gaps in the literature regarding the role of MMP/TIMP dysregulation in CVeD pathophysiology. Specifically, the aim was to take into consideration two other variables: TGFβR expression within the vein wall and CVeD clinical progression. Moreover, unlike the majority of the studies in this field, two methodological measures were taken for the purpose of controlling additional sources of variability. Firstly, specimens were grouped and compared based on anatomic harvest site (evidence that vein source and location may be a factor in the variability has been shown previously [19]), and secondly, comparison groups were matched regarding important demographic and clinical features.

The choice of specific MMPs was based on previously published studies [29], yet only genetic data concerning *MMP2*, *MMP9*, *MMP12*, *TIMP1*, *TIMP2*, *TIMP3*, *TIMP4*, *TGFβR2*, and *TGFβR3* are discussed as RT-PCR results showed no detection of *MMP8* and *MMP13* gene expression. Likewise, qPCR results obtained from cDNA pools of CVeD and healthy veins showed negligible *MMP1* and *TGFβR1* gene expression. The absence of *MMP8* and *MMP13* gene expression in vein samples contradicts previous findings [19,28,33]. *MMP8* is frequently associated with venous ulcer healing [34,35]; however, it may not intervene in venous wall restructuring.

In our study, gene expression of *MMP12*, *TIMP2*, *TIMP3*, *TIMP4*, and *TGFβR2* was decreased in CVeD veins (especially in early clinical stages) when compared to healthy veins. Elevated gene expressions of *MMP2*, *TIMP1*, and *TIMP3* in varicose veins were previously described [23,25], while decreased expression of *MMP2* was also reported [18,24]. Methodological differences—for example, sample size, anatomic harvest site or use/non-use of effective control samples—may partially explain the conflicting results. The disparity in genetic data among studies may also be due to the broad range of morphologic presentations of varicose vein walls (atrophic or hypertrophic segments), and the possible existence of different phases in MMP and TIMP expression/activity throughout CVeD progression. It is plausible that the imbalance of these proteins favors atrophy during a primary phase and fibrosis during a secondary phase. In view of this, vein specimens at different CVeD stages were stratified for comparison in the current study.

Significant differences were only found between healthy and CVeD veins (i.e., these differences were not present between CVeD vein groups from the tibiotarsal junction—cf. Figure 2 and Figure 3). Despite this, we believe that in a larger sample the trends presented in Figure 3 may achieve statistical significance. We also believe that these trends give a good representation of what happens in CVeD atrophic/hypertrophic phases: a local decrease in MMP and TIMP gene expression in varicose veins from the CEAP2–3 group (during the atrophic phase) followed by a local gradual increase in MMP and TIMP gene expression in varicose veins from the CEAP4–6 group (during the hypertrophic phase). Regarding TGF-β1 receptors, decreased gene expression of the signal transducer *TGFβR2* in varicose veins could suggest a counter-regulation mechanism to control chronically elevated local levels of TGF-β1, leading to a reduction in participation of this growth factor in the MMP/TIMP imbalance throughout CVeD clinical progression. This is in line with previous studies advocating a correlation between TGF-β1 enhanced expression/activity and the development of varicosities [10,12,36,37,38]. Although TGF-β1-enhanced expression was previously found in varicose veins [36,37], its signal transducer receptor expression has not been extensively studied [39]. However, whilst our results for TGFβR gene expression may explain generally decreased gene expression of MMP and TIMP in CVeD veins (especially from early clinical stages) when compared to healthy veins, they do not explain the slightly increased gene expression of MMP and TIMP in the CEAP4–6 group (when compared to the CEAP2–3 group). It might be that another inflammatory cytokine/growth factor (e.g., interleukins, vascular endothelial growth factor, or tumor necrosis factor-α) [40,41] may play a role in linking pressure-induced leukocyte infiltration, vein wall inflammation, and alteration in MMP and TIMP expression/activity during the CVeD hypertrophic phase.

Before discussing further results regarding CVeD clinical progression, it should be noted that genetic data across anatomical vein regions were uneven (Figure 3) and this may be important. If molecular events are not uniform in the venous system, measures of MMP and TIMP expression/activity should always be reported with reference to vein region and comparisons among vein specimens harvested from different anatomical regions might not be reliable.

Only gene expression of *MMP2*, *MMP9*, *TIMP3*, and *TGFβR3* presented differences between CVeD groups. On the one hand, *MMP2* and *TIMP3* gene expressions were increased in advanced CEAP stages (from tributary veins); on the other hand, *MMP9* and *TGFβR3* gene expressions were decreased in advanced CEAP stages (from the saphenofemoral junction and tributary veins, respectively).

MMP2 and MMP9 have been long recognized as major contributors to proteolytic degradation of ECM [42]. Contrary to others’ findings [20,23,26,43], no significant differences in gene expression of both gelatinases were found between healthy and CVeD veins, which is most probably due to the sample size. However, *MMP2* and *MMP9* gene expression seemed to evolve differently throughout CVeD clinical progression. This might be due to distinct response processes to different inflammatory cytokines/growth factors (other than TGF-β1) during the hypertrophic phase. Also, MMP9 (unlike MMP2) might have its preponderant proteolytic role in early CVeD stages rather than in advanced stages. This is coherent with previous studies in which an increase in plasma pro-MMP9 activity (but not MMP2) was found in response to 30 min postural blood stasis in patients with varicose veins [21]. Nevertheless, the only significant result for *MMP9* was achieved in veins from the saphenofemoral region and this region might not be as reliable as the others for CVeD group comparisons (as proximal and distal segments of veins may be affected differently by the disease [19]).

The decrease in *TGFβR3* gene expression throughout CVeD clinical progression is consistent with our previous supposition. If a counter-regulation mechanism to control chronically elevated local levels of TGF-β1 was in place, this coreceptor (whose main function is to regulate TGF-β1 binding and signaling through its corresponding receptors) [5,44,45] may be part of the mechanism.

With respect to IHC results, it should be explained that the selection of proteins submitted to this technique was a consequence of previous qPCR results obtained from CVeD and healthy veins: it was assumed that the proteins with higher gene expression levels (MMP2, TIMP2, and TGFβR2) were most likely to present immunostaining differences. Our results showed that MMP2, TIMP2, and TGFβR2 can be detected mainly in the tunica intima and media of healthy and varicose vein walls, although in a lower quantity in the latter. Lower levels of MMP2, TIMP2, and TGFβR2 in varicose veins, when compared to controls, have been partially described by others [18,24] and are in accordance (particularly regarding tunica intima) with our qPCR results for similar comparisons (CVeD groups vs. control group).

Regarding IHC results between the CVeD groups, it is worth mentioning that these were not always coherent among anatomical regions, suggesting once more that comparisons among vein specimens harvested from different locations might not be reliable. Nevertheless, intima layer results (from all anatomical regions) were, in general, consistent with our qPCR results. MMP2 presence in all tunicae was mainly unchanged between CVeD groups with one exception: at the saphenofemoral junction where a slightly lower presence was found in the CEAP4–6 group. This is coherent with the trend revealed with the qPCR results (Figure 3). In tributary veins, MMP2 presence was generally unchanged between the two groups, while the qPCR results showed an increase in the CEAP4–6 group. This may be explained by the subjective nature of IHC results. With regard to TIMP2 presence, the results are generally in line with Figure 3 trends, particularly for the tunica intima. Finally, the equally low presence of TGFβR2 across CVeD groups was consistent with our qPCR findings, reinforcing the idea of a counter-regulation mechanism to reduce local TGF-β1 expression or signaling throughout CVeD clinical progression.

Whilst it has been suggested that MMP/TIMP imbalance could potentially work through elements (especially smooth muscles cells) in the tunica media [46], we highlight the importance of the tunica intima in CVeD pathophysiology, as shown by the coherence found between qPCR and IHC results for this tunica (in vein specimens from all anatomical regions). It was also noted that among the three anatomical vein regions, varicose tributary veins showed more evident differences (especially in qPCR results) between the CVeD groups, probably due to its thinner media. A thinner media tunica may make the venous walls more vulnerable to homeostatic upset induced by local hypertension, and therefore more prone to varicosity and premature morphologic changes.

## 4. Materials and Methods

### 4.1. Specimen Collection

Samples (2 cm length) of healthy great saphenous veins were harvested from the tibiotarsal junction of 13 patients undergoing coronary bypass surgery (control group). Samples of pathologic refluxing great saphenous veins (from the saphenofemoral and tibiotarsal junctions) and varicose tributaries (from veins showing tortuosity and significant diameter increase with blood filling at the thigh or leg), including the adventitia, were obtained from 31 patients submitted to surgical ablation of the great saphenous vein (CVeD group). The methods of harvesting, storage, and processing samples were identical in both groups.

Before collection, preoperative venous duplex ultrasonography was performed (to confirm venous reflux in CVeD samples and its non-existence in controls) and CVeD patients were evaluated according to the CEAP (Clinical, Etiologic, Anatomic and Pathophysiologic) classification [47] and then regrouped (CEAP2–3/CEAP4–6 groups). Subjects with the following conditions were excluded: surgery within the previous six weeks; steroids or intravenous drug use; deep vein thrombosis or thrombophlebitis; active infection; and collagen diseases and conditions that could modify leukocyte activity (e.g., diabetes mellitus, neoplasia, rheumatoid arthritis, vasculitis). After collection, all vein samples were aseptically washed free of blood using a physiological salt solution, immersed in RNA-Later (Ambion^®^, Carlsbad, CA, USA), refrigerated at 4 °C for 24 h, then snap frozen in liquid nitrogen and stored at −80 °C until use.

In compliance with the Declaration of Helsinki, all the procedures carried out with human samples were approved by the Ethics Committee of “Cova da Beira University Hospital Centre, Covilhã, Portugal” (protocol No. 28/2009, 26 February 2009). Informed consent was obtained from all participants.

### 4.2. Conventional and Quantitative Real-Time Polymerase Chain Reaction

Total ribonucleic acid (RNA) was isolated from 50 to 100 mg of tissue sample using the TRI Reagent (Ambion^®^, Carlsbad, CA, USA) and following the manufacturer’s instructions. For complementary deoxyribonucleic acid (cDNA) synthesis, 500 ng of total RNA was reverse transcribed using the M-MuLV Reverse Transcriptase kit (NZYTech^®^, Lisbon, Portugal) in a final volume of 20 mL. Both procedures have been described elsewhere [30].

To confirm the gene expression of *MMP1*, *MMP2*, *MMP8*, *MMP9*, *MMP12*, *MMP13*, *TIMP1*, *TIMP2*, *TIMP3*, *TIMP4*, *TGFβR1*, *TGFβR2*, and *TGFβR3* in CVeD and healthy vein samples, conventional polymerase chain reactions (PCR) were performed using the NZYTaq DNA polymerase kit (NZYTech^®^, Lisbon, Portugal) in accordance to the manufacturer’s instructions. PCR reactions were carried out in a final volume of 25 µL containing 1 µL synthesized cDNA, 0.1 µL Taq DNA polymerase (5 U/µL), 0.625 µL deoxyribonucleoside triphosphate solution (10 mM), 1.2 µL sense and antisense primers (5 pmol/µL) for each gene (STABVIDA^®^, Lisbon, Portugal; Table 3), 1.5 µL MgCl_2_ (50 mM), 16.875 µL nuclease-free water, and 2.5 µL 10× Taq DNA polymerase buffer. After initial heating at 95 °C for 5 min to denature the cDNA, 35 cycles (30 cycles for β-actin) of 30 s at 95 °C, annealing 30 s at 60 °C, and extension 30 s at 72 °C were carried out. The final cycle was followed by a period of 5 min at 72 °C to ensure that the amplified DNA was double-stranded. The integrity of cDNA samples was assessed by amplification of the β-actin housekeeping gene. The PCR products were electrophoresed on a 1% agarose gel and visualized with GreenSafe (NZYTech^®^, Lisbon, Portugal) using UV light.

The gene expression of positively-confirmed MMP, TIMP, and TGFβR was determined by quantitative real-time PCR (qPCR) using gene-specific primers (STABVIDA^®^, Lisbon, Portugal; Table 3) and SYBR-Green/Fluorescein qPCR Master Mix (Fermentas Life Sciences^®^, Vilnius, Lithuania). β-actin housekeeping gene was used to normalize gene expression levels. The efficiency of the amplifications was determined for all primer sets using serial dilutions (1, 1:5 and 1:25) of cDNA. Primer concentrations and annealing temperatures were optimized, and the specificity of amplicons was determined by melting curve analysis.

The qPCR was performed as described elsewhere [30].

Both conventional and quantitative real-time PCR was carried out for a pool of specimen cDNA and then for each specimen separately.

Fold differences were calculated using the formula 2^−ΔΔ*C*t^ [48].

### 4.3. Immunohistochemistry

Vein specimens were fixed in 2% paraformaldehyde/0.2% glutaraldehyde for 24 h, transferred to a 70% alcohol solution and paraffin embedded the following day. Paraffin-embedded vein blocks were cut into 6 mm sections and mounted onto poly-l-lysine-coated slides.

Immunostaining of MMP2, TIMP2, and TGFβR2 was performed according to an optimized immunohistochemistry (IHC) protocol. Paraffin-embedded vein sections were deparaffinized, rehydrated, and pre-treated with Trilogy^TM^ solution (Cell Marque^®^, Rocklin, CA, USA). Endogenous peroxidase activity was blocked by incubation in 3% (*v*/*v*) H_2_O_2_ for 10 min. Before and after incubation steps, the sections were washed twice for 5 min with Tris-buffered saline containing 0.1% Tween^®^-20 (Rockford, IL, USA) (TBS-T) at room temperature. Vein sections were incubated for 1 h with primary polyclonal rabbit antibodies against human MMP2 (1:700 dilution; ab38917, Abcam^®^, Cambridge, UK), TIMP2 (1:500 dilution; ab74216, Abcam^®^, Cambridge, UK), and TGFβR2 (1:500 dilution; ab61213, Abcam^®^, Cambridge, UK) at room temperature. A goat biotinylated anti-rabbit IgG (Vector Laboratories^®^, Burlingame, CA, USA) was used as secondary antibody. The specificity of the staining was accessed by omitting the primary antibody. After 10 min amplification and 10 min detection steps, the sections were then incubated in peroxidase substrate solution (diaminobenzidine) in a dark chamber for 10 min, counterstained with Mayer’s hematoxylin for 3 min, rinsed for 10 min, and mounted for observation under a Zeiss LSM-710 laser scanning confocal microscope (Carl Zeiss, Oberkochen, Germany).

Staining was developed for the same period of time for each antibody, for both control and CVeD specimens, and was scored in a blinded manner by two independent observers. The final results took into account the staining intensity and relative difference between different groups of vein specimens (control, CEAP2–3, and CEAP4–6).

### 4.4. Statistical Analysis

Statistical analysis was performed by means of IBM SPSS Statistics, (v.22.0, Armonk, NY, USA). Using data from subsamples (selected by quota sampling) of the 44 participants, statistically significant differences in gene expression among three (controls vs. CEAP2–3 vs. CEAP4–6) or two (CEAP2–3 vs. CEAP4–6) groups of participants were tested for. ANOVA (followed with Bonferroni tests) or the Student test was used to compare the means for independent groups. Equivalence between the groups, regarding participants’ demographic and clinical features, was assessed using Fisher’s exact test or its Freeman-Halton extension (for 2 × 3 contingency table) and Student test or ANOVA, as appropriate. To meet parametric assumptions, data were transformed using log_10_(x) when necessary and outliers were controlled. Before violation of those assumptions, non-parametric tests (Kruskal–Wallis test, followed by the Mann-Whitney test) were performed. Data descriptive statistics are presented as (absolute and relative) frequencies, mean values ± standard error of the mean (SEM) and ranges. All tests were two-tailed and the significance was set at *p* ≤ 0.05.

## 5. Conclusions

Whilst further studies about molecular events in varicose vein walls are required, our results have contributed more evidence on MMP/TIMP imbalance in venous walls throughout CVeD clinical progression, as well as on the role of TGF-β1 in this event. Differences in MMP and TIMP expression should be expected not only among healthy, atrophic, and hypertrophic varicose veins, but also across anatomical vein regions. The full functional role of TGF-β1 remains to be defined but our results regarding TGFβR expression may suggest a counter-regulation mechanism to control chronically elevated local levels of TGF-β1, leading to a reduction in participation of this growth factor on MMP/TIMP imbalance throughout CVeD clinical progression.

These findings represent another step towards the understanding of CVeD pathophysiology and may provide some cues for therapeutic approaches targeting TGF-β1 signaling.

## Figures and Tables

**Table 1:** Participants’ demographic and clinical features. Data shown in this table refer to the total number of participants from which subgroups were selected (in order to achieve demographic and clinical feature equivalence) for further analyses.

Features		Control	CEAP2–3	CEAP4–6
Sex	Females	3 (23.08%)	14 (73.68%)	9 (75%)
	Males	10 (76.92%)	5 (26.32%)	3 (25%)
Age (a)		67.85 ± 2.679 (54–81)	56.37 ± 1.764 (40–74)	59.58 ± 2.930 (45–77)
BMI (kg/m^2^)		25.28 ± 0.935 (20.89–29.07)	28.26 ± 1.072 (22.83–37.46)	28.82 ± 1.232 (23.15–35.55)
Pregnancies (No.)		2.33 ± 1.856 (0–6)	2.07 ± 0.322 (0–4)	2.89 ± 0.351 (2–5)
CEAP	2	-	2 (6.45%)	-
	3	-	17 (54.84%)	-
	4	-	-	10 (32.25%)
	5	-	-	1 (3.23%)
	6	-	-	1 (3.23%)

**Table 2:** Immunodetection of MMP2, TIMP2, and TGFβR2 in tissue sections of healthy, CEAP2–3, and CEAP4–6 veins (from three different regions). The scores were established in a blinded manner by two independent observers, according to the labeling observed with a microscope, as follows: −, negative; +, discrete; ++, moderate and +++, intense.

Region	Tunica	Group	MMP2	TIMP2	TGFβR2
Tibiotarsal junction	Intima	Controls	+++	++	+
		CEAP2–3	+	−	−/+
		CEAP4–6	+	+	+
	Media	Controls	+++	++	+
		CEAP2–3	+	+	−/+
		CEAP4–6	+	+	−
	Adventitia	Controls	+++	−	−/+
		CEAP2–3	−	−/+	−
		CEAP4–6	−	−	−
Saphenofemoral junction	Intima	CEAP2–3	++	+/++	+
		CEAP4–6	+	+	+
	Media	CEAP2–3	++	+/++	−/+
		CEAP4–6	−/+	+	−/+
	Adventitia	CEAP2–3	−	++	−
		CEAP4–6	−	−/+	−
Tributary	Intima	CEAP2–3	+	−	−/+
		CEAP4–6	+	−/+	−
	Media	CEAP2–3	+	+	−/+
		CEAP4–6	−	−/+	−/+
	Adventitia	CEAP2–3	−	−/+	−
		CEAP4–6	−	−	−

**Table 3:** Oligonucleotide sequences and amplicon sizes in conventional and quantitative real-time PCR. bp: base pairs.

Gene	Primer Sequence (5′–3′)	Amplicon Size (bp)
β-actin	Sense: CAT CCT CAC CCT GAA GTA CCC	202
	Antisense: AGC CTG GAT AGC AAC GTA CAT G	
TIMP1	Sense: GAC GGC CTT CTG CAA TTC C	79
	Antisense: GTA TAA GGT GGT CTG GTT GAC TTC TG	
TIMP2	Sense: GAG CCT GAA CCA CAG GTA CCA	77
	Antisense: AGG AGA TGT AGC ACG GGA TCA	
TIMP3	Sense: CCA GGA CGC CTT CTG CAA	73
	Antisense: CCC CTC CTT TAC CAG CTT CTT C	
TIMP4	Sense: CAG CCT CAG CAG CAC ATC TG	75
	Antisense: GGC CGG AAC TAC CTT CTC ACT	
MMP1	Sense: AAG ATG AAA GGT GGA CCA ACA ATT	70
	Antisense: CCA AGA GAA TGG CCG AGT TC	
MMP2	Sense: AAC TAC GAT GAC GAC CGC AAG T	142
	Antisense: AGG TGT AAA TGG GTG CCA TCA	
MMP8	Sense: CAC TCC CTC AAG ATG ACA TCG A	135
	Antisense: ACG GAG TGT GGT GAT AGC ATC A	
MMP9	Sense: AGG CGC TCA TGT ACC CTA TGT AC	111
	Antisense: GCC GTG GCT CAG GTT CA	
MMP12	Sense: CGC CTC TCT GCT GAT GAC ATA C	136
	Antisense: GGT AGT GAC AGC ATC AAA ACT CAA A	
MMP13	Sense: AAA TTA TGG AGG AGA TGC CCA TT	124
	Antisense: TCC TTG GAG TGG TCA AGA CCT AA	
TGFβR1	Sense: ACG GCG TTA CAG TGT TCT G	358
	Antisense: GGT GTG GCA GAT ATA GAC C	
TGFβR2	Sense: GCA GGT GGG AAC TGC AAG AT	76
	Antisense: GAA GGA CTC AAC ATT CTC CAA ATT C	
TGFβR3	Sense: CTG TTC ACC CGA CCT GAA AT	502
	Antisense: CGT CAG GAG GCA CAC ACT TA	
